# Length of Stay in the Emergency Department during COVID-19 Pandemic in a Tertiary Care Hospital: A Descriptive Cross-sectional Study

**DOI:** 10.31729/jnma.6281

**Published:** 2021-05-31

**Authors:** Suraj Singh, Bibek Koirala, Rabin Thami, Anupama Thapa, Bijay Thapa, Anuj Kayastha, Priyanka Dahal

**Affiliations:** 1 Kanti Children's Hospital, Maharajgunj, Kathmandu, Nepal

**Keywords:** *COVID-19*, *emergency department*, *length of stay*, *pandemic*

## Abstract

**Introduction:**

Emergency Department overcrowding has become worsening problem internationally which may affect patient, emergency department efficiency and quality of care and this may lead to increased risk of in-hospital mortality, higher costs, medical errors and longer times to treatment. With this pandemic COVID-19 likely to go on for months, if not a year or longer, the Emergency Department should be prepared for large influx of patients infected with COVID-19. The aim of this study is to find-out the length of stay in emergency department during COVID-19 pandemic at a tertiary care hospital in Nepal.

**Methods:**

This is a descriptive cross-sectional study conducted in the Emergency Department of Kanti Children's Hospital. Ethical clearance was obtained from Institutional review committee-Kanti Children's Hospital. Data collection was done from the emergency records from July 23, 2020 to July 29, 2020. The calculated sample size was 211. The data thus obtained was entered in Statistical Package for the Social Science software version 20 and necessary calculations were done.

**Results:**

The median length of stay in emergency department was found to be 1.75 hours (Interquartile range 0 to 30 hours).

**Conclusions:**

Definitive management starts in respective wards and Intensive Care Units. During COVID-19, with longer emergency stay, chances of cross-infection increases, and the health workers serving in emergency department will be at risks. So guidelines for shorter emergency stay should be implemented.

## INTRODUCTION

With the declaration of COVID-19 as a pandemic by the World health organization, emergency department (ED), intensive care unit (ICU) practitioners, hospital administrators, governments, policymakers must prepare for the surge in critically ill patients.^1,2^ But the health system in Nepal has never been geared to face an onslaught of infectious disease.^3^

Overcrowding has always been the major issue in ED which is due to an imbalance between patient input, ED throughput and patient output and has a negative impact on patient's health.^4-8^ Especially delay in diagnostic testing along with numbers of beds on isolations wards and ICU are the biggest concerns.^9^ As the dedicated tertiary children's hospital in Nepal, we were at the epicentre of this pandemic and particularly affected.

The main aim of this study is to find out the median length of stay (LOS) in ED during COVID-19 pandemic.

## METHODS

This is a descriptive cross-sectional study conducted in Kanti Children's Hospital, Nepal. Data was taken from July 23, 2020, to July 29, 2020. Ethical clearance was obtained from the Institutional review committee - Kanti Children's Hospital. All the patients presenting in the emergency department during this period having complete data were included in the study. The patients with incomplete or missing data were excluded from the study.

Convenient sampling was done and the sample size was calculated as,


$$\begin{array}{ll} \text{n} & \text{= }{\text{Z}}^{\text{2}}\times {\text{σ}}^{\text{2}}\;/{\text{e}}^{\text{2}}\\ & \text{= }{\left(1.96\right)}^{\text{2}}\times {\left(2.5\right)}^{2}/{(\text{0.5)}}^{\text{2}}\\ & \text{= }96.04\\ & =96 \end{array}$$

Where,

n = minimum required sample size
Z = 1.96 at 95% Confidence Interval (CI)
σ = Standard Deviation taken from a previous study, 2.5 hours^10^
e = margin of error, 0.5 hour

Since the convenient sampling technique was used, the sample size was doubled to 192. Also, considering 10% of records with missing data the sample size was 211.

Emergency stay duration was calculated as the time in between the presentation of the patient till the patient leaves the ED. The result of the stay in this study comprises of observation stay, discharged cases, discharged on request (DOR), referral, leave against medical advice (LAMA), brought dead and death in ED, admission to the isolation ward and suspected COVID ICU, admission to various ICU i.e. NICU and SICU, admission to burn isolation ward. The data was entered in Statistical Package for the Social Sciences (SPSS) version 20 and required descriptive statistics were calculated.

## RESULTS

During COVID-19, the median length of emergency stay was found to be 1.75 hours with minimum stay of 0 hour to maximum stay up to 30 hours as shown in (Table 1).

**Table 1:** Length of Stay in Emergency Department (in hour)

Duration	Median	n	Minimum	Maximum	Mode
During COVID-19 Pandemic	1.75	211	0	30	0.25

The maximum percentage of patients i.e. 18 (8.5%) of the patients stayed for 0.25 hours in emergency department. Among the patients who presented to ED, majority were male during COVID-19 duration as shown in (Table 2).

**Table 2:** Sex Prevalence.

Duration		Frequency n (%)
**During COVID-19**	Male	143 (67.8)
	Female	68 (32.2)
	Total	211 (100.0)

The result of stay with their median length of stay in emergency department during COVID-19 are as shown in (Table 3).

**Table 3:** Result of stay.

**Duration of stay in Emergency during COVID-19 (in hour)**				
Result of stay	Median	n	Minimum	Maximum
**Observation**	0.63	92	0.00	11.25
Discharge	2.75	43	0.00	23.33
DOR	18.50	4	3.33	21.00
Referral	2.50	8	0.50	9.50
LAMA	2.08	3	0.75	2.58
Brought Dead	1.67	1	1.67	1.67
Admission	3.5	60	0.23	30
Total	1.75	211	0.00	30

**Figure 1.**
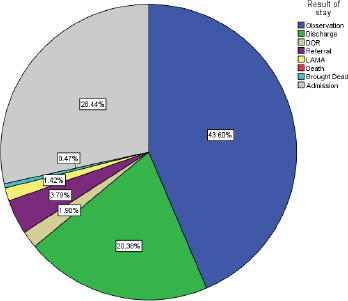
Result of stay during COVID 19.

Among the patient admitted from ED during COVID-19, 43.60% were kept in observation which is followed by admission to the wards. The discharge percentage of the patients from ED was found to be 20.38 % during COVID-19 Pandemic (Figure 1).

**Figure 2.**
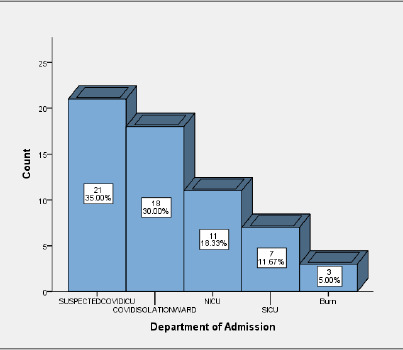
Department of admission during COVID-19.

During COVID-19 pandemic, among the patients admitted from ED, 21 (35%) patients were admitted in Suspected COVID ICU followed by 18 (30%) patients admitted in COVID isolation ward, 11 (18.33%) patients in NICU, 7 (11.67%) patients in SICU, 3 (5%) in burn isolation ward as shown in figure 2 (Figure 2).

Among the admitted patients, the median length of ED stay according to various department wise during COVID-19 are shown in (Table 4). The median LOS in ED among the patient requiring admission in the Burn ward remains the shortest.

**Table 4:** Department of admission.

Duration of stay in Emergency during COVID-19 (in hour)				
**Department of Admission**	**Median**	**n**	**Minimum**	**Maximum**
NICU	2.42	11	0.23	30.00
SUSPECTED COVID ICU	3.50	21	0.50	9.25
COVID ISOLATION WARD	4.13	18	0.25	11.50
SICU	2.67	7	1.00	22.25
Burn	0.75	3	0.25	0.75

## DISCUSSION

Only a few studies regarding the LOS in the emergency department during the COVID-19 pandemic has been conducted worldwide. However, there has not been such a study conducted in Nepal on the emergency stay duration during the COVID-19 pandemic, so we have tried to find out the median length of emergency stay during this pandemic.

Previous studies conducted during pre-COVID-19 duration by Lamsal, et al. showed a median of emergency length of stay as 3.84 hours with a minimum of 0.4 hours and maximum of 87.21 hours of stay.^11^ In a study done by Chrusciel, et al. the median LOS was 3.58 hours before the intervention and 3.10 hours after implementation of fast track in emergency department.^12^ Likewise a study done by Hofer, et al. the median length of emergency stay was found to be 2.06 hours.^13^

The median LOS in ED during COVID-19, which was found to be 1.75 hours with a minimum of 0 hour to maximum of 30 hours in our study. The median LOS in ED during COVID-19 found in our study is less as compared to the study done study of Lamsal et al, Chrusciel et al and Hofer, et al. In our study, the maximum percentage of patients i.e. 8.5 % of the patients stayed for 0.25 hours in emergency department.

The difference in the findings of ED stay among the studies can be due to the variation in patients flow because of lockdown, hospital setup with the availability of resources and also due to difference in ED protocols. Among the patients presenting in paediatrics emergency, some of them don't have to stay in emergency making their stay duration as 0 hour because they actually do not require any intervention apart from good counselling with explanation of danger signs. They are just brought to paediatrics emergency because of parental fear and anxiety. In most cases, vulnerable child syndrome has been noticed.

The patients requiring admissions in isolation ward or intensive care units stayed longer in ED as compared to the ones who were directly discharged from the ED or kept in observation. This may be due to age factor, delay in consult request, late ward and ICUs around, complicated cases, insurance support, unavailability of the diagnostic test during off-hours, lack of unoccupied beds in the isolation ward and intensive care units. When patients spend a lot of time in the ED, their inpatient evaluation and treatment doesn't typically start until they get to a respective ward; so the delays in the treatment they experience might contribute to longer overall hospitalization for these patients.^14^

The number of patients requiring monitoring in ICU during a pandemic is high as shown by our study. The patients requiring admission was initially admitted to the isolation ward and later trans-out to the respective department's ward, once after their report of real time polymerase chain reaction for SARS-COV2 come negative. If the report of patients came positive, then all the health workers who were directly in contact with the patients were quarantined. And this guideline issued by the hospital administration has reduced the chance of cross-infection among the health workers and patients and ultimately leading to effective efficiency of the hospital during the outbreak.

New insights can be generated from this study to reduce LOS. The number of COVID-19 cases is increasing day by day, so there is a need for the establishment of a well-designed and responsive emergency care system throughout Nepal. The concerned authorities should be more focused on the uniform implementation of guidelines for emergency care during health crisis such as COVID-19 outbreaks to decrease morbidity, mortality and financial burden among the patients visiting ED. There are no such studies that describe this trend in more detail, so more study needs to be done to find out the factors influencing the length of stay in ED in such a Pandemic.

Since this research was conducted in one centre, it cannot reflect the generalized scenario of the ED stay of the other healthcare setting of country and also the data are regenerated reviewing the ED record book. The study is of short duration, it may not completely picture the LOS and outcomes in other duration of pandemics when the patient's flow is very high or low. Hence, the result lacks external validity.

## CONCLUSIONS

Patients spending a shorter duration in emergency especially during pandemic outbreak have an overall good prognosis. Also, it will reduce the chance of crosscontamination. The emergency stay can be shortened more if scientific guidelines are implemented and adequate resources for case management are available in health care centres throughout Nepal.
